# Phosphorus Cycling in Montreal’s Food and Urban Agriculture Systems

**DOI:** 10.1371/journal.pone.0120726

**Published:** 2015-03-31

**Authors:** Geneviève S. Metson, Elena M. Bennett

**Affiliations:** 1 Department of Natural Resource Sciences, McGill University, Sainte Anne de Bellevue, Montreal, Quebec, Canada; 2 McGill School of Environment, McGill University, Montreal, Quebec, Canada; Duke University, UNITED STATES

## Abstract

Cities are a key system in anthropogenic phosphorus (P) cycling because they concentrate both P demand and waste production. Urban agriculture (UA) has been proposed as a means to improve P management by recycling cities’ P-rich waste back into local food production. However, we have a limited understanding of the role UA currently plays in the P cycle of cities or its potential to recycle local P waste. Using existing data combined with surveys of local UA practitioners, we quantified the role of UA in the P cycle of Montreal, Canada to explore the potential for UA to recycle local P waste. We also used existing data to complete a substance flow analysis of P flows in the overall food system of Montreal. In 2012, Montreal imported 3.5 Gg of P in food, of which 2.63 Gg ultimately accumulated in landfills, 0.36 Gg were discharged to local waters, and only 0.09 Gg were recycled through composting. We found that UA is only a small sub-system in the overall P cycle of the city, contributing just 0.44% of the P consumed as food in the city. However, within the UA system, the rate of recycling is high: 73% of inputs applied to soil were from recycled sources. While a Quebec mandate to recycle 100% of all organic waste by 2020 might increase the role of UA in P recycling, the area of land in UA is too small to accommodate all P waste produced on the island. UA may, however, be a valuable pathway to improve urban P sustainability by acting as an activity that changes residents’ relationship to, and understanding of, the food system and increases their acceptance of composting.

## Introduction

People have significantly altered the P biogeochemical cycle, changing P flows between ecosystems [1], modifying the geographic distribution of P stocks around the world [2], and greatly accelerating the global P cycle [3]. Global P cycling naturally happens on geological time scales, where P is eroded from rocks, tightly recycled through ecosystems, eventually ending up in the ocean where it is reincorporated into sediments [4]. People have accelerated the extraction process through mining to produce P fertilizer for agricultural systems [5], roughly tripling the mobilization of P at the global scale [1]. Although fertilizer use has markedly improved crop productivity, it has led to increased losses of P to waterways from agricultural landscapes that in turn threaten important aquatic resources with overfertilization [6].

Anthropogenic changes to the P cycle pose a two-sided problem. On the one hand, we face scarcity of non-renewable mined-P resources [7,8], with a limited amount of concentrated P deposits [9] geopolitically concentrated in a few countries [10]. Three countries (Morocco, China, and the USA) control 93% of the currently known mineable resource [11,12]. Because there are no known substitutes for P in agriculture, the high levels of current P extraction create concern for future food security. On the other hand, P losses from agricultural and urban ecosystems to aquatic ones through runoff and erosion have led to eutrophication in many lakes and coastal ecosystems [13,14]. The number of hypoxic water bodies around the world have been increasing, threatening ecosystem health, water quality (affecting drinking water supply as well as recreation) and fisheries on which we depend [15]. Current management of P resources is thus both a threat to future food security and to the downstream ecosystems on which we depend for a multitude of ecosystem services. Solutions to both problems are related—the less P is wasted or lost to downstream ecosystems, the more P is available for use elsewhere and in the future [16].

Understanding urban P cycling is a key component in understanding anthropogenic P cycling at regional and global scales [17]. Cities drive the production of high P-products through consumption (including human and pet foods, landscaping and gardening materials, timber products and construction and materials), and produce high-P waste (human excreta, and food and landscaping waste). As such, cities are linked to agricultural and other ecosystems through trade, as well as through hydrological and atmospheric dispersion patterns. Such linkages make cities part of problematic P management, but also key to finding solutions. In fact, cities are often centers of creativity and innovation, and as such altering natural resource management within cities can have large effects at larger geographical and political scales [18]. Developing a conceptual and empirical understanding of urban P cycling is thus a key part of understanding global P cycling and of finding solutions to problematic P management locally and globally.

In order to transform cities from centers, or hotspots, of P cycling to ecosystems that contribute to sustainable P management, we also require better information about the real potential and feasibility of proposed solutions in specific cities. One proposed solution is the use of urban and peri-urban agriculture to recycle urban P-waste back into food production at the local scale. [19] acknowledge the lost resources (including nutrients and water) urban agriculture (UA) can utilize, and studies about UA in Ghana and Ethiopia have highlighted its role in addressing both food security and sanitation issues through nutrient recycling [20]. However, the majority of studies have not quantitatively examined UA from a nutrient perspective with city-specific data. In order to glean answers to questions about the potential and feasibility of UA as P management strategies we need to examine the current use of UA practices. We must first quantify how we currently manage P with location specific-data, and then evaluate how we can manage P more sustainably in the city.

Understanding the role of UA in P recycling is particularly important in cities where new urban planning and management documents are changing to include sustainability goals. Montreal (Quebec, Canada) has adopted a new organic waste management policy and is experiencing growing public, government, and private support for UA, which could impact P cycling in the city. The Quebec provincial government has mandated that 100% of all organic waste (green waste, food waste, and sewage) be recycled by 2020. Assuming that compost products are applied to agricultural land, this increase in organic waste recycling would translate into P recycling. At the same time, there has been increasing public support for UA, culminating in 29,000 signatures on a petition asking the municipality for a formal public consultation process on UA in Montreal in 2011 [21]. In response, the Montreal municipal government created a permanent committee on UA, bringing together key government, non-governmental organizations, private companies, and academics to jointly advise the city on how best to support current UA projects and their the expansion In addition, the Conférence régionale des élus de Montréal (CRÉ) has adopted a plan to guide Montreal in the development of a sustainable and equitable food system. One of the plan’s core themes is to reduce the ecological footprint of the city’s food system through measures that include increasing food waste recycling and increasing local production in UA [22].

**Fig 1:**
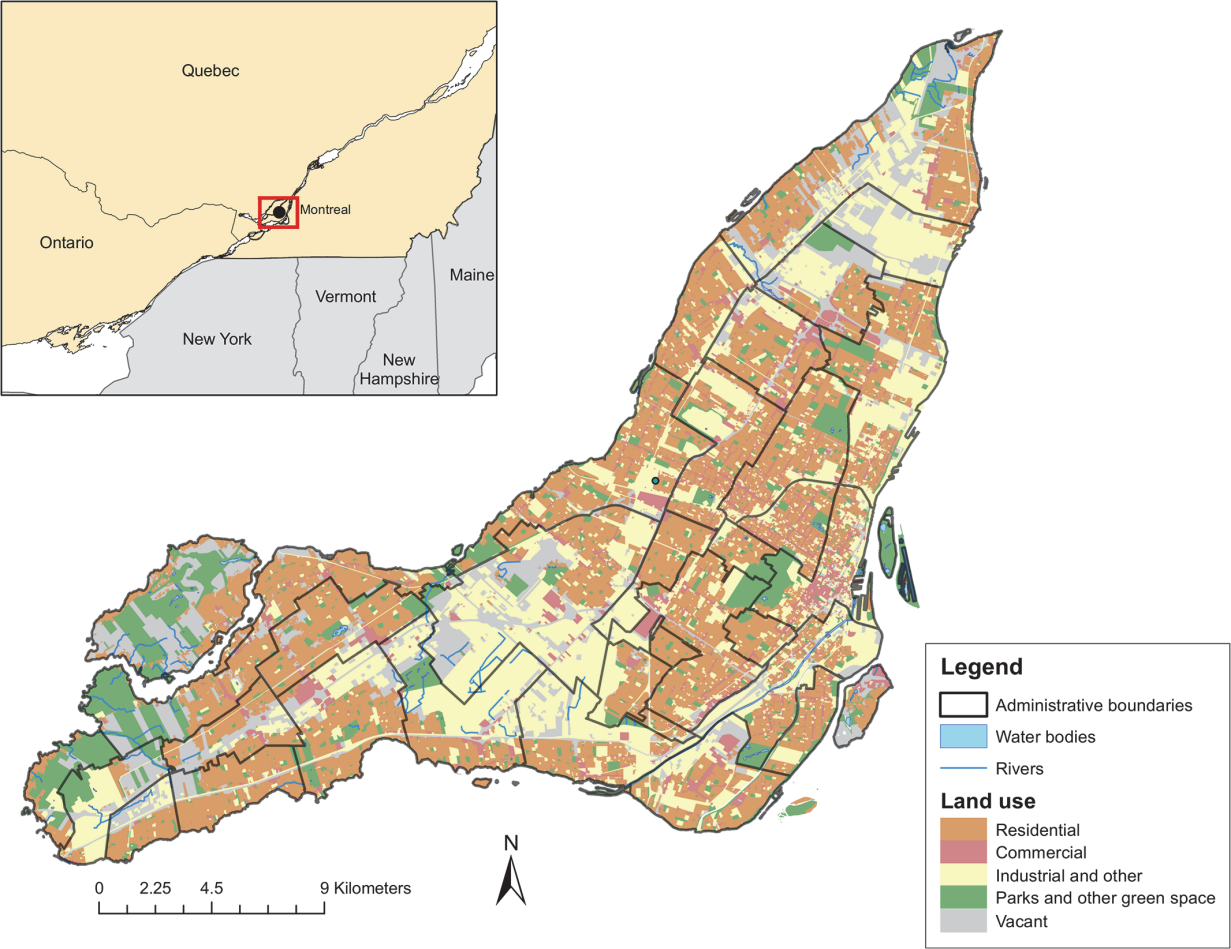
Montreal island geographical situation and land uses. The island of Montreal is aproximately 38% residential, 12% green space, 14% vacant lots, and 18% industrial and commercial land uses. Residential land-use includes high, medium, and low density housing, commercial land use includes malls, service-industry buildings, and business district, Industry and other land use includes light and heavy industry, quarries, public and education institutions, landfills, and service utility areas, Parks and other green space land use includes golf courses, cemeteries, regional and city parks, natural reserves, and rural sites [23]. Municipalities and borough limits are indicated by the black administrative boundaries.

However, we do not know how much P is currently cycling (or being recycled) through the food and waste system of Montreal, or through the UA system on the island. Such benchmark information is essential to understand how policies and practices in Montreal may change P cycling in the future. Here, we aim to better understand the current and potential role of UA in urban P cycling and recycling by examining two key systems in urban P cycling on the island of Montreal, Canada (Fig.1). We quantify P movement through the Montreal island food system (which we define as all food imported and consumed, and all food and sewage waste produced on the island), and the UA system (which we define as the fertilizers imported, crops harvested, animals raised, and organic waste produced through UA on the island).

## Methods

### P flow calculation

**Fig 2:**
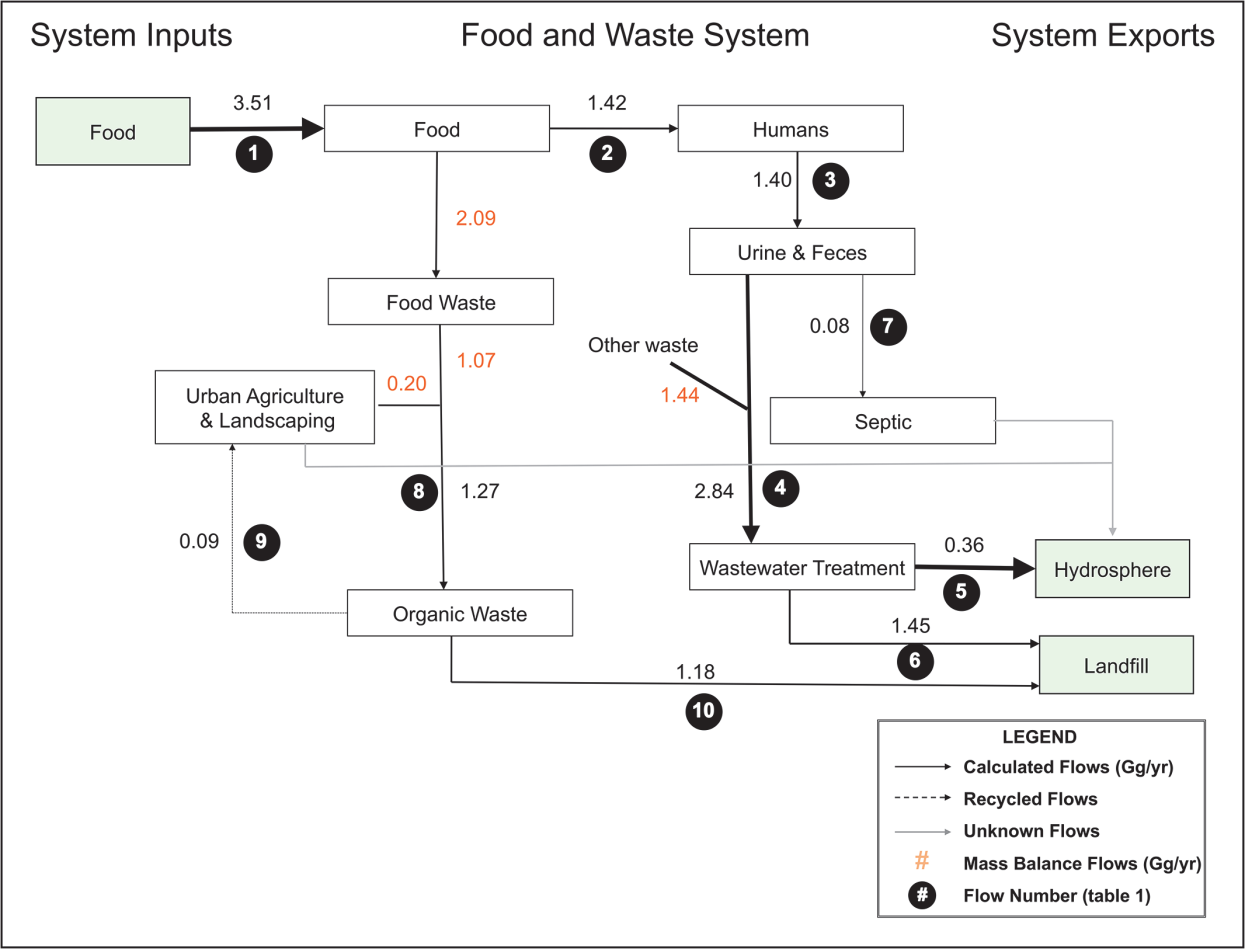
Phosphorus flows in the food system on the island of Montreal in gigagrams of P yr^-1^ where the size of arrows represents the magnitude of flows. Recycled flows are represented by dashed arrows, unknown flows are represented by grey arrows, and flows calculated by mass balance (subtracting or adding calculated flows) are represented by orange numbers. Green boxes represent inputs and exports to and from the island. Numbers in black circles represent the flow identification number, which is associated with a description of the flow and calculation methods in Table 1.

**Fig 3:**
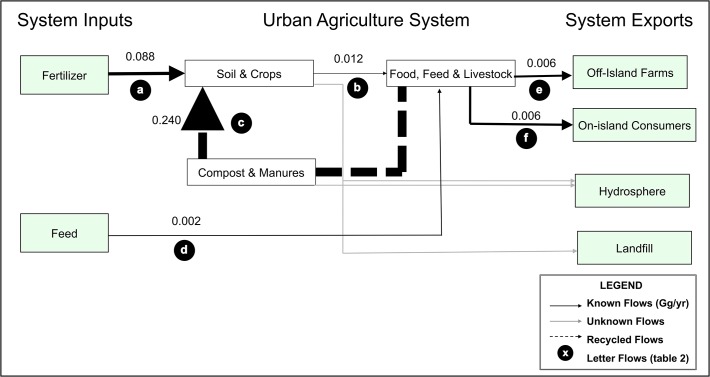
Phosphorus flows in the urban agriculture (UA) system on the island of Montreal in gigagrams of P yr^-1^ where the size of arrows represents the magnitude of flows. Recycled flows are represented by dashed arrows, and unknown flows (i.e., runoff and erosion to the waterways, and amount of organic material from UA sent to landfill) are represented by grey arrows. Green boxes represent inputs and exports to and from the UA system. Letters in black circles represent the flow identification letters, which are associated with a description of the flow and calculation methods in Table 2.

We used substance flow analysis (SFA, [24]) to quantify P flows for the year 2012 in two separate systems on the island of Montreal: the food system (Fig. 2), and the UA system (Fig. 3). Montreal Island (approximately 500 km^2^, population 1.98 million in 2012) is located in the Saint-Lawrence River [23]. Because the food system and the UA system have unique P flows, we completed separate data collection and flow calculations for each of the two systems. Our analysis of the food system focuses on flows of P onto and off of the island in food and organic waste, while our analysis of the UA system focuses on the use and sources of P for UA on the island of Montreal. Each flow, in both systems, was calculated by multiplying the weight of the material by its P concentration.

To calculate P flows through the Montreal food system we quantified P in food imports to the island (1), food consumed on the island (2), human urine and feces produced on the island (3), sewage waste going to the wastewater treatment plant (4), sewage treatment plant losses to the Saint-Lawrence river (5), biosolids sent to landfill (6), septic storage (7), food and green waste produced on island (8), food and green waste produced recycled through compost (9), and food and green waste produced sent to landfill (10, numbers refer to Fig. 2 and Table 1). We considered both food and green organic waste in the calculation of flows 7, 8, and 9 because the City waste management department does not differentiate them in their reports and yet we wanted to use this data as it is the most accurate site-specific information possible. The P concentrations for flows were found in published literature and government reports, and quantities (mass) were obtained through official government reports (see Table 1 for the equations and a full list of data sources and assumptions used to calculate the P flows considered in the food and waste system). Because different data sources were used to calculate each P flow, some discrepancies between inputs, outputs and wasted P are present in our study of the Montreal food system. We used site-specific information whenever possible, with regional or national averages to supplement site-specific information as needed.

**Table 1:** Data sources for Montreal food system P budget.

**Flow number (in Fig. 2)**	**Flow name**	**Equation**	**Data sources**	**Assumptions and specifications**
**1**	**P imports in Food**	(Food supply* P concentration of food* population)– (percentage pre-market food wasted* food supply* P concentration of food*population)+ (2*Restaurant and industry organic waste)	P concentration of food: [25,26], [27], Population: Satistics Canada (2013), Pre-market food waste: [28], [29,30], Restaurant and industry organic waste: Solinov (2012), Fortin et al. (2011)	Food imports were based on Montreal’s total population in 2012 and FAO average Canadian diet, both in terms of content and quantities. FAO reports diet in quantities grown, not eaten, thus quantities were transformed based on average North American food waste percentages before reaching retail stores. Because this was based on resident population, we added the food entering the system through restaurants and industry. We had information on organic waste produced by restaurants and industry, and the percentage of food wasted, but not food imports. As such we back-calculated food imported by using the percentage wasted (50%) and the amount. We only included food entering the city for consumption and ignored food products that transit through the city to be exported elsewhere, and as such we are looking at the net import and export of P in the Montreal food system.
**2**	**Food P consumption**	P imports in food (Flow 1)–(Post-market food waste * P concentration of food waste)	Post-market food waste: [28], [29,30]P concentration of food items: [25,26], [27]	Food consumption was calculated by subtracting the estimated amount of food wasted before it is consumed (thus including waste at stores and at home) from the food entering the island.
**3**	**P excreted**	Flow 2 * percentage excreted	Percentage of P excreted by humans: [31]	
**4**	**P entering wastewater treatment plant (WWTP)**	(Water entering plant * P concentration in water entering) + (biosoilds to landfill * P concentration of biosolids)	Volume of water entering plant: [32] in m^3^ yr^-1^, P concentration in water entering: [32] in mg of P l^-1^, Biosolids to landfill: [32] in dry matter (DM) tons yr^-1^, P concentration of biosolids: Personal communication with sewage treatment plant expressed in %P^2^O^5^ DM	Montreal has only one wastewater treatment plant on the island. The quantity of water and P concentration of that incoming water, as well as the amount of biosolids collected by the plant and their P concentration were used to calculate the total P entering the plant.
**5**	**P leaving WWTP to water**	Water leaving plant * P concentration in water leaving	Volume of water leaving plant: [32] in m^3^ yr^-1^, P concentration in water leaving: [32] in mg of P l^-1^	The quantity of water and P concentration of that outgoing water from the plant were available through official reports and used to calculate the total P leaving the plant.
**6**	**Biosolids P entering landfill**	Biosoilds to landfill * P concentration of biosolids	Biosolids to landfill: [32] in dry matter (DM) tons yr^-1^, P concentration of biosolids: Personal communication with sewage treatment plant expressed in %P^2^O^5^ DM	The treatment plant currently incinerates all biosolid waste and sends it to landfill, and we used the amount of biosolid ash and its concentration in P to calculate the total P going to landfill. However, we did not include P that may be found in the sands used in the water treatment process at the plant and subsequently landfilled or P in the large residues collected at the plant because of lack of data.
**7**	**P entering soils through septic system**	Boisolids produced in septic system * P concentration of biosolids	Biosolids produced in septic system: [33,34] in %P^2^O^5^ in DM	Although most of the island is connected to the WWTP, there still are some septic systems. We used official government data on the amount of biosolids produced by septic systems on the island in 2001, thus assuming that any population growth on the island happened in areas connected to the WWTP. We used a biosolid P concentration reported for average municipal sewage waste because a concentration was not available for septic systems in the province of Quebec.
**8**	**Organic waste (food and green waste) P produced**	(Residential organic waste recycled * inverse of percentage of organic waste recycled* proportion of organic waste that is food* food waste composition*P concentration in food waste) +(Residential organic waste recycled * inverse of percentage of organic waste recycled* proportion of organic waste that is green* P concentration in green waste) + (Business organic waste * P concentration of food waste)	Residential organic waste recycled and population served: Ville de Montreal (2013a) in kg person^-1^ yr^-1^ and % of total organic waste recycled, [25,26,35–37] [38,39]014), Buisness organic waste produced: Solinov (2012) in tons yr^-1^	We calculated the amount of P in organic waste (food waste, green landscaping waste, and wood) generated on the island by using official government estimates of organic waste recycled by residents, businesses, and institutions, and back-calculating to the total waste produced based on the percentages recycled. Proportion of organic waste that was food versus green waste was determined through communication with the City waste department, based on their internal data We included green and wood waste even though they are not strictly part of the food system as they are used in most compost and thus tested P contents reflect the inclusion of such waste products. We used P contents for fruits and vegetables (for food), green waste, and wood according to their proportional make-up of waste. The P concentrations include the conversion to dry weight.
**9**	**Organic waste P recycled**	(Residential organic waste recycled * proportion of organic waste that is food* P concentration in food) +(Residential organic waste recycled* proportion of organic waste that is green* P concentration in green waste) + (Business organic waste recycled * P concentration of food)	Organic waste recycled and population served: Ville de Montreal (2013a) in kg person^-1^ yr^-1^ and % of total organic waste recycled, [25,26,35–37] [38,39]014)	We calculated the amount recycled through composting using both official government figures of organic waste currently recycled through households (11%) and adding the amount of organic waste recycled of businesses known to compost. Here we use the average fruit and vegetable P concentration instead of weighting by Canadian food waste make-up because the city doesn’t currently compost high amounts of meats and processed foods.
**10**	**Organic waste P landfilled**	Flow 8- Flow 9		

We did not include runoff and erosion losses, or P lost in storm events due to wastewater treatment plant limited capacity to treat the high volume of water produced during these storm events because of a lack of data.

To calculate P flows through the UA system (that which produces food, feed, and pasture for livestock on the island), we quantified the following, where the letters refer to the symbology used in Fig. 3 and Table 2: P in fertilizer imports (a), harvested crops (b), compost and manure reused on the island (c), imported feed and animal supplements (d), food and feed exported (e), and food from local UA production consumed on the island (f). We surveyed local practitioners to get information on the area under production, the type of substrate used, the type and quantity of P applied to farms and gardens, the amount of harvested crops and animal products, and the organic waste recycled or leaving the system. We determined whether the P flows entering and leaving the UA system (referred to as a budget) were balanced (with inputs equaling outputs), were accumulating (inputs exceeding outputs, causing the system to accumulate P), or depleting (outputs larger than inputs of P).

**Table 2:** Description of flow calculations for urban agriculture P budget.

**Flow letter** (in Fig. 3)	**Flow name**	**Equation**	**Assumptions and Specifications**
**a**	P fertilizer and soil amendments imported applied soil	Sum for all gardens in type n [(total P inputs from off-island source/ area of garden)*(area of garden/total area of UA type n surveyed)] estimated area for type n	Weighted P application by area of farm or garden, and by the estimated area for the 3 types of management, so type n is type of management (see x, y, z). See Table 3 for types of inputs considered
**b**	P in harvested crops (feed and food)	Sum for all gardens in type n [(total P harvested/ area of garden)*(area of garden/total area of UA type n surveyed)] *estimated area for type n	Weighted P application by area of farm or garden, and by the estimated area for the 3 types of management, so type n is type of management (see x, y, z)
**c**	P compost and manure from on-island sources applied to soil	Sum for all gardens in type n [(total P inputs from on-island sources/ area of garden)*(area of garden/total area of UA type n surveyed)] *estimated area for type n	Weighted P application by area of farm or garden, and by the estimated area for the 3 types of management, so type n is type of management (see x, y, z). We combined recycled inputs (plant residues, compost, vermicompost, and animal manures) into one flow in order to maintain anonymity of survey respondents
**d**	P imported as animal feed and supplements	Sum for all types [(Feed or supplement imported type n*P concentration type n)]	Did not scale to estimated area of UA because we surveyed all known farms that raise animals and P concentrations were obtained by survey respondents or by manufacturers
**e**	P exported off island (food, feed, and manure)	P as exported manure + P as exported feed	Did not scale to estimated area of UA because we surveyed all known farms that export
**f**	P consumed by on-island residents	(P harvested—P harvested for animal feed) + P in animal products (milk and eggs)	P harvested is scaled to total UA area but P in animal feed and P in animal products are not because we surveyed all known farms that raise animals
**x**	** Estimating total area: UA private and community garden type	(% of households practicing UA* % of practicing households doing UA in back-, side-, front-yard *# of households on island * average size of vegetable garden)+ (% of households practicing UA* % of practicing households doing UA on roof or balcony*# of households on island * area of 4 alternatives containers (0.96m^2^))+ (area of community gardens)	References: Household participating in UA: [41], Area of private backyard gardens: [42], Community garden area: [21]
**y**	Estimating total area: UA collective garden type	(Area surveyed collective gardens)+(area of missing collective gardens with known area)+(average area of known collective gardens reporting area*# of collective gardens with unknown area)	Reference: Area of collective gardens not surveyed: [43]
**z**	Estimating total area: UA farm type	Known area of farms from survey + reported area of the 2 farms we did not survey	Reference: Area of farms not surveyed: [43]

Data are from surveys, and if P content was not provided by the survey respondent values in Table 3 were used. Note that we did not include flows relating to runoff and erosion losses or inputs from soil and soil mixes if P content was not available from the survey respondent (e.g., soil, potting-mix, vermiculite, perlite, or coco fiber).

### Urban agriculture system data collection and processing

To obtain quantitative data on P flows and information on general nutrient management practices, we conducted in-person surveys with commercial farmers (10 surveys in total), private and community gardeners (83), and organizations managing collective, institutional, and work-place gardens (50) between April and November 2013. We scaled these survey results by the estimated area under UA production to calculate the overall P budget for the UA flows on the island of Montreal. McGill University Research Ethical Board approved the protocol for administering the survey, survey questions, and data management and storage protocols (REB File # 995-0213). Written consent was obtained from participants whenever possible through signature, although oral consent was also approved, and was documented by the researcher checking the consent box on the survey form (see S1 Text for additional information on survey administration, sampling strategies, and specific survey questions).

To best sample all types of UA on the island we first separated UA practitioners into three categories based on the size of the agricultural operation and the type of management: 1) farms, which included for-profit enterprises and large-scale university farms, 2) collective, institutional, and business gardens, which included gardens where many individuals may participate in the gardening, but decisions about fertilization, management, and harvest are made collectively or centrally by an organization or agronomic advisor, and 3) community and private citizen gardens, where each individual gardener makes decisions about his/her plot of cultivated land. We used different sampling strategies for these three categories. For farms and collective gardens, we developed an initial list of UA practitioners to survey [21] and used the snowball method [40] to ensure we had contacted as many relevant actors as possible. This method entails asking respondents to suggest (or recruit) other relevant actors that we should survey until we have surveyed (or tried to survey) all the actors mentioned (i.e., no or few new actors are mentioned at the end of the survey process). The large number of community and private gardens, and lack of comprehensive public registry, necessitated more opportunistic sampling of this group. For community gardens, we communicated with garden presidents to gain access to the garden area and then completed surveys on-site with gardeners that agreed to meet with us. We were successful in gaining access to at least one community garden site in each of 13 boroughs (out of a total of 19 city boroughs). For private gardens, we contacted possible respondents through electronic mailing lists of city gardeners and then used snowball sampling to find additional potential respondents, ultimately completing 33 surveys.

Some conversions and assumptions were necessary to transform survey answers into P flows at the garden scale and to calculate P flows for the island as a whole. Table 2 describes P flow calculations and assumptions, and Table 3 describes data sources for density of materials, dry matter content, and P content used when site-specific information was not available (see S1 Text for more information on data processing, including how we estimated yield when this information was not available through a survey; S1 Fig. for a comparison of known UA yields to those used in this study; and S1 Table for the collected data).

**Table 3:** Data (and data sources) used to calculate P inputs when they were not available with information directly from survey.

**Inputs**	**Specification**	**Bulk density**	**Dry Matter**	**P content**	**Data sources, assumptions, and specifications**
Vermicompost		600 kg m^3–1^		0.0115 P conversion	[44]
Shrimp and/or crab compost (or other marine based compost)		0.41507 kg l^-1^		0.75% P_2_O_5_	Average based on the commercial fertilizers found in Montreal hardware and garden stores that had information on density or P content
Bio-forest compost		.41666 kg l^-1^		0.8% P_2_O_5_	Used numbers on Fafard company bio-forest compost bags found in stores
Plant-based compost (green and table waste)		533.8783 kgm^3–1^	30%	1%P	Bulk density is average of “good compost” according to [45], home compost west island [46] for DM and P content because they are specific to Montreal. Compost bulk density varies from 700–1,200 pounds per cubic yard, and desirable is consider 800–1000 pounds per cubic yard
Sheep/goat manure	non-composted		28%	4lbs P_2_O_5_ ton^-1^	[47]
	composted	0.417 kg l^-1^		0.4% P_2_O_5_	Used numbers on Signature master gardener brand bags found in stores
Cow/beef manure	composted	12.5 kg bag^-1^ (assume its 30l bag but that is not explicitly stated)		0.4% P_2_O_5_	Used average of values for brands found in store
Chicken manure (including quail)	litter	546.5 kg m ^3–1^		1.538% P	[48]
	composted	10 kg 30l bag^-1^		3% P_2_O_5_	Used numbers on Actisol brand bags found in stores
Horse manure	non-composted		46%	4 lbs ton^-1^	[47]
	composted	NA	45%	0.3% P_2_O_5_	Used numbers on Solabiol brand (found online December 2013 http://www.solabiol.com/nos-solutions/planter/les-amendements-pour-fertiliser/fumier-de-cheval)
Pig manure	liquid	1 kg l^-1^		0.9kg 1000l^-1^	[49,50]
Liquid fertilizer		1 kg l^-1^			Assumed density of water
Bone meal		1 kg l^-1^		10% P_2_O_5_	Commercial inputs found in stores didn’t report both density and P content so assuming 1 to 1 ratio (and online values very but are close), P concentration is average of what was reported in stores
Shrimp and/or crab meal (or other marine based meal)				3.5% P_2_O_5_	Used numbers on Bionord brand bags found in stores
Fish emulsion		1 kg l^-1^		4% P_2_O_5_	Used numbers on Acadie brand bottles found in stores and assuming density of water
Marine algae		1.0007 g ml^-1^		1.5% P_2_O_5_	Used the average of brands found in stores
Straw		150 kg m^3–1^	88%	0.08375% P (DM basis)	Density if for a little rectangle bail in Quebec with medium packing in [51], DM is average of straws listed in [52], P content is an average of [52], [53], [54]
Hay		150 kg m^3–1^	0.4209	0.2987% P	
Wood chips				0	Assuming 0 for hard dry woods (see BRF for younger wood)
Leaves		163.15 kg m^3–1^	39%	0.1015% P	Middle point between high valued of uncompacted leaves and low point of compacted leaves according to: [55],for DM [56] P value is middle point of the leaf litter values found in [57] (used this number over yard waste because dead leaves don’t contain as much as fresh ones)
Rameal frangmented wood (BRF)		492 kg m^3–1^ (fresh density)	65.35%	0.26% P_2_O_5_ (DM basis)	[58]
Lawn and yard waste		577.257487kg m^3–1^		0.3% P	Density [39], P content [38]
Grass				0.3% P	P content [38]
Potting mix and fertilized potting mix (e.g. miracle grow mix)				0	Because P in soil and potting mixes is not systematically reported, we did not include them in P inputs except when site-specific information was available. We did however include the use of soils in our count of types of inputs used.
Black soil		0.291 kg l^-1^		0	Non-weighted average of all soils that were commercially available and had both weight and volume on the bag
Peat				0	[59]
Perlite				0	[59]
Vermiculite				0	[59]
Coco fibers				0	[59]
Crop yield		0.643 kg m^3–1^		0.0003 P and DM conversion	Weighted average of yields in Montreal gardens by area [60], and New York city community gardens with tomatoes [61], P content is average of fruits and vegetables as used in [62]. See S1 Fig. for more detail on yield assumptions

We estimated the total area under UA production on the island of Montreal to scale our survey results, and thus P flows, to the whole Montreal UA system (see Table 2 for the equations and data sources used to estimate the total UA area on the island and Table 4 for information on the proportion of the total UA area we surveyed). (See S1 Text for more detailed instructions on how we estimated the total area in UA production, as well as assumptions used to calculate P flows in the UA system).

**Table 4:** The proportion of total area sampled for each of three types of UA found on the island of Montreal.

**Type of social organization managing UA**			
**Type**	**Number of respondents**	**Total area surveyed in km** ^**2**^ (% of total)	**Total area estimated to be cultivated on the island in km** ^**2**^ (% total)
Community and personal gardens	83	0.001 (0.04%)	13.9 (77.34)
Collective, school, business, and institution gardens	50	0.02 (0.74%)	0.03 (0.15)
Commercial farms (and large university farms)	10	3.10 (99.21%)	4.05 (22.5)
Total	143 (665 gardens)	3.12	18.00
**Substrate type in UA**			
**Type**	**Number of respondents**	**Total area surveyed in km** ^**2**^	**n/a**
Soil (on the ground)	89	3.1	
Both soil and container	28	0.002	
Containers and roof top	26	0.008	

### Future scenarios

To determine how UA might contribute to recycling municipal P in Montreal and how it might help Quebec meet its provincial goal of diverting 100% of organic waste form landfills, we calculated the following:

1. The amount of P that could be recycled if all P applied to the current area in UA originated from on-island recycled sources.
2. The amount of area in UA needed to recycle all P in the food and yard waste currently produced on the island.
3. The amount of area in UA needed to recycle P in food and yard waste if organic waste production decreased by 50%.

In all scenarios we assume a sustainable situation in which P budgets are balanced (no accumulation or depletion where we consider the P requirements per m^2^ are the same as in the current state).

## Results and Discussion

### P cycling in the Montreal food system

In 2012, 3.51 Gg P (flow 1) were imported to the island of Montreal in food, 0.36 Gg P (flow 5) was exported in wastewater to the Saint-Lawrence river, and 2.63 Gg P (flow 6+10) were exported to landfills (Fig. 2). The majority of P entering the island system ultimately accumulated in landfills because the majority of solid organic waste (89%, flow 9 in proportion to flow 8) and all incinerated biosolids from the wastewater treatment plant were disposed of in this manner. A small amount of sewage waste was treated by septic systems and this P was considered to be stored in the ground on the island (0.08 Gg, flow 7). Some organic waste was composted or left on the soil (0.09 Gg P, flow 9, representing six percent of current P wasted as food or green materials).

### P cycling in the Montreal UA system

We now move from the food system for the entire island to the smaller Montreal UA system, which accumulated P with a soil P surplus of 0.316 Gg P yr^-1^ (flows a+c-b). That is, UA practitioners harvested less P in food and feed (0.012 GgP yr^-1^ flow b) than was applied to soils in 2012 (0.328 Gg P yr^-1^ flow a+c, Fig. 3). Ultimately, only 27% (flow a proportion of flows a+c) of P inputs applied to garden and agricultural soils were imported to the island, while the vast majority (73%, flow c proportion of flows a+c) came from on-island sources, including green-waste compost, vermicompost, and manure. Of the P harvested in crops grown on the island, 48% was consumed on the island as food (fruits, vegetables, milk, and eggs, part of flow f as a proportion of flows f+e), 22% was consumed as feed on island (part of flow f as a proportion of flows f+e), and 28% was exported (mostly as soy and corn, part of flow e as a proportion of flows f+e). Excess manure (48% of manure produced on island) was exported to off-island farms (part of flow e as a proportion of flows f+e, see Table 2 for data sources and equations).

P cycling in Montreal’s UA system is only a very small part of the overall food system of Montreal. P inputs to the UA system (fertilizers and feed) are only 2.58% of the P imported to Montreal in the larger food system (comparing flow 1 to flow a). P in crops, milk, and eggs produced on the island through UA represent only 0.43% of the P in food distributed to people through grocery stores and restaurants (comparing flow 2 to flow f).

### Potential for UA to recycle more P from the food system

Two ways to increase P recycling on the island are to increase the percentage of P inputs to UA from on-island recycled sources and to expand the area under cultivation. Increasing the percentage of recycled P inputs to UA is possible but is unlikely to play a large role in augmenting overall P recycling in Montreal, as UA practitioners already use recycled P for 73% of their fertilizer applications. However, a phone survey by the city of Montreal on UA practices indicated that only 23% of Montreal UA practitioners composted [41]. P recycling may thus not be as high as our survey results indicate, and changes in nutrient management practices would be needed to ensure high P recycling in these gardens. In addition, increasing yields, and thus P uptake, could increase the potential demand for recycled P in UA.

**Fig 4:**
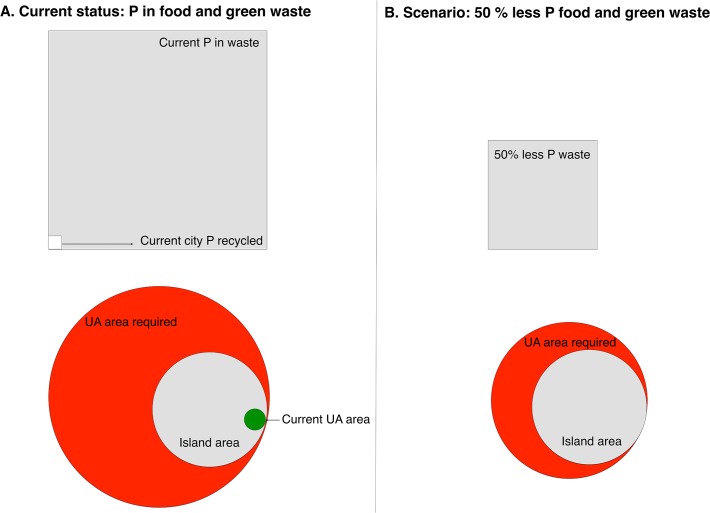
Current and potential future role of urban agriculture (UA) in Montreal P cycling. Two scenarios are visually represented in terms of amount of P waste produced, recycled, and the amount of corresponding land in UA production required. Panel A represents current state of P in food and green waste, the amount of UA, and the area necessary to recycle all that waste through UA. The amount of P in food and green waste is represented by the grey square on the top left, the small white square represents the proportional amount of the P wasted that is currently recycled, represents that estimated amount of P recycled through UA. The grey circle represents Montreal island area, and the green circle the proportional area of the island under UA production. The large red circle illustrates the area of UA necessary to recycle all P waste produced (317% bigger than the island) is P application is equal to P harvest and all P application is from recycled sources. Panel B illustrates the area of UA required (186% bigger than the island, represented as the red circle) if Montreal produces 50% less P as food and green waste (all symbols are proportional in size to those in Panel A).

Increasing the area under cultivation would increase the amount of P recycled; however, it could not recycle all P used on the island. We estimated that it would require 1850 km^2^ of UA (an area nearly four times larger than the island of Montreal) to utilize all P waste currently produced (Fig. 4). Even if Montreal were to produce 50% less P as organic waste (via increasing efficiency in the food system), the area needed for UA to utilize all waste P would still be almost twice the area of the island. Thus, assuming current yields and balanced P application, UA cannot effectively recycle all P outputs from the Montreal food system. Partnerships with off-island peri-urban farms could increase recycling of local P and thus help the city meet the 100% organic waste recycling by 2020 goal set by the provincial government. If Montreal increased cultivated area seven-fold, to a total of 25% of the total island area, we would only recycle a limited 6.8% of the P in the island’s food and yard waste (assuming P application rates that meet crop needs where additional UA land comes from converting 27% of low and medium density residential and 10% of all public green space like parks).

Increases in UA production could be facilitated by current public support and government policies for UA, but it is important to note that such land is in competition for multiple uses. Montreal has put in place a food system plan [22], and created a permanent committee on UA [21] in order to support current UA and increase it. The potential of low-technology farming on vacant lots, as well as high-technology farming on rooftops, to produce all of the vegetables needed for Montreal demonstrates that UA can indeed be an important contributor to achieving Montreal’s more sustainable and equitable food system goal [63]. Although UA may provide multiple benefits, there are competing uses and priorities for all of these spaces within the planning context [64].Increases in real-estate prices for example could decrease the amount of vacant land and increase building density, making less space available for UA, and thus affecting its capacity to recycle P. In addition, environmental contamination and possible health risks associated with food production in urban environments could limit expansion (as studied in [65–67].

### Potential of UA and cities in sustainable P management

Although we may not be able to dramatically increase P recycling directly through increases in UA area or amount of recycled P used in UA, UA may still be able to play an important indirect role in by encouraging more recycling of P. For example, urban farms and gardens can act as educational spaces for people to learn about food production and nutrient cycling [64,68,69], which could increase knowledge about composting and advance the social acceptability of large scale composting and reuse of urban waste. As a space where the dependence of cities on agricultural production is visible. UA may be part of how urban populations learn about more sustainable food systems, including nutrient recycling [70,71].

This paper has focused on the quantitative role UA can play in recycling P in food and green waste in Montreal, but other increases in P use efficiency and recycling pathways are possible. Changing diets by decreasing meat consumption [62], as well as decreasing food waste by retailers and consumers [72,73] can significantly decrease the amount of P required to grow food for Montreal, entering the island, and ultimately ending up in landfills and the Saint-Lawrence river. P from wastewater treatment plants could be recycled back into agricultural production (including UA) if properly treated to remove pathogens as well as organic and inorganic contaminants, including heavy metals [72]. Biosolids and wastewater can recovered and reused on agricultural lands and act as a recycling pathway for P as has been done in many cities in developed (e.g., Phoenix USA [74]) and developing countries (e.g., Kumasi Ghana [20]). In summary, Montreal has many ways to increase P use efficiency and recycling, and UA may be able to facilitate some of these changes, even if its quantitative role in recycling is limited.

## Conclusion

Issues of P scarcity and P pollution make sustainable P management a pressing issue from the global to the local scale. Cities have an important role to play in increasing P sustainability as they concentrate both P demand and waste production. UA has the potential to contribute to sustainable P management by facilitating the reuse of waste P in nearby gardens and farms. We used the island of Montreal as a case study to investigate the current P recycling in the food system and UA system to examine how much of this potential is used. In Montreal, the current P food system is dependent on imported food, and the majority of P waste is stored in landfills, with only a very small amount of P recycled as compost. UA is a small part of P cycling in Montreal’s overall food system. Even though the majority of P inputs to UA come from recycled sources, the food and feed harvested still only represents only a small fraction of Montreal’s overall P demand. Although UA in Montreal could not possibly recycle all P from on-island organic waste, UA may still have potential to increase P recycling, primarily by acting as an activity that changes residents’ relationship to, and understanding of, the food system and increases their acceptance of composting [75]. This study serves as a quantitative benchmark to understand P cycling in the food and UA systems, and to monitor the effect of changes in policies and practices over time in Montreal. As key ecosystems on our landscape, understanding and monitoring nutrient cycling in cities is necessary for sustainable resource management, and our Montreal case study demonstrates that although cities have potential to recycle P internally through UA and back to peri-urban agricultural land, this potential is not always fully utilized.

## Supporting Information

S1 Fig Average and range (high and low) of yields reported in UA studies compared to our study. In this study we used a weighted average (by area) of data reported in [60] and the highest reported value in in [61] (because it included tomatoes and mixed vegetables). Montreal reported yields came from 8 gardeners (38 gardens, [60]), New York yields reported in [61] came from data in community gardens and urban farms, and Oakland California yields are from [67] estimating possible yields based on conventional agriculture yields and low and medium biointensive cultures. “From our surveys” are the average, maximum, and minimum values for the 37 participants that had yield data, nine of which were farms. And the “survey exc. Farms” represents the average, maximum and minimum values in the collective, private and community gardens we surveyed.
(DOCX)

S1 Table Detailed anonymous response data on phosphorus application, harvest, and waste management for collective, private, business, and community urban agriculture respondents in Montreal. (XLSX)

S1 Text Methods (DOCX)
