# Supplementary material for: Phosphorus Cycling in Montreal’s Food and Urban Agriculture Systems
Source: PLoS One. 2015 Mar 31;10(3):e0120726. doi: 10.1371/journal.pone.0120726 (PMC4380336; doi:10.1371/journal.pone.0120726)
Supplement: S1 Fig — In this study we used a weighted average (by area) of data reported in [60] and the highest reported value in in [61] (because it included tomatoes and mixed vegetables). Montreal reported yields came from 8 gardeners (38 gardens, [60]), New York yields reported in [61] came from data in community gardens and urban farms, and Oakland California yields are from [67] estimating possible yields based on conventional agriculture yields and low and medium biointensive cultures. “From our surveys” are the average, maximum, and minimum values for the 37 participants that had yield data, nine of which were farms. And the “survey exc. Farms” represents the average, maximum and minimum values in the collective, private and community gardens we surveyed. (DOCX) [file pone.0120726.s001.docx]

Figure S1. Average and range (high and low) of yields reported in UA studies compared to our study. In this study we used a weighted average (by area) of data reported in Duchemin et al. (2009) and the highest reported value in in Ackerman (2011) (because it included tomatoes and mixed vegetables). Montreal reported yields came from 8 gardeners (38 gardens, Duchemin et al. (2009)), New York yields reported in Ackerman (2011) came from data in community gardens and urban farms, and Oakland California yields are from McClintock et al. (2013) estimating possible yields based on conventional agriculture yields and low and medium biointensive cultures. “From our surveys” are the average, maximum, and minimum values for the 37 participants that had yield data, nine of which were farms. And the “survey exc. Farms” represents the average, maximum and minimum values in the collective, private and community gardens we surveyed.
