# Supplementary material for: Phosphorus Cycling in Montreal’s Food and Urban Agriculture Systems
Source: PLoS One. 2015 Mar 31;10(3):e0120726. doi: 10.1371/journal.pone.0120726 (PMC4380336; doi:10.1371/journal.pone.0120726)
Supplement: S1 Text — (DOCX) [file pone.0120726.s003.docx]

**S1 Text: Methods**

- 1. **Survey administration**

Our survey consisted of eight primary questions with a four additional questions about animal production when relevant. The survey used 2012 as a reference year for larger gardens and farms that kept records. If records did not exist for 2012, which was the case for most small and private gardeners, we used 2013 as a reference. When ever possible, surveys were conducted in the garden or in the office where quantitative data were stored. We measured the area of gardens ourselves when the participant did not have such records. We counted the number of bags or containers of different types of fertilizer and nutrient inputs and noted the weight and NPK ratio of all inputs when it was accessible. We also asked for copies of any supporting documentation. All survey data were entered and stored in through Limesurvey online system [1], and was done with McGill Research Ethical Board approval (REB File#: 995-0213).

Following are the specific research questions asked:

1. Indicate the beginning of the growing season (first time plants are planted) and the end of the growing season (last harvest) in the garden (s) with an "x".

| **Month** | **Week 1** | **Week 2** | **Week 3** | **Week 4** |
| --- | --- | --- | --- | --- |
| January |  |  |  |  |
| February |  |  |  |  |
| Mach |  |  |  |  |
| April |  |  |  |  |
| May |  |  |  |  |
| June |  |  |  |  |
| July |  |  |  |  |
| August |  |  |  |  |
| September |  |  |  |  |
| October |  |  |  |  |
| November |  |  |  |  |
| December |  |  |  |  |

1. How much area does each of the following types of gardens does your organization/institution/company manages?

|  | **Area** | **Measurement unit a) square meters; b) square feet; c) acres; d) % of garden(s);**  **e) other (specify)** | **Additional information** |
| --- | --- | --- | --- |
| **Total area** (all types of gardens together) |  |  |  |
| **In soil** (directly in Montreal earth or in raised beds) |  |  |  |
| **In containers** (includes all pots, smart pots, biotops, and any other container that is of a “movable size”) |  |  |  |
| **On roof** (gardens directly on the roof with a member and NOT in containers) |  |  |  |
| **Hydroponics** (without soil) |  |  |  |
| **Other** (specify) |  |  |  |

* We define containers as an object that can easily be moved

3. What percentage of the total space of the garden is used for food production? This **includes**: planted area, alleys to move around, and orchards. It does **not include**: play and other recreational areas, grassy areas, compost production area, or storage area.

|  | % |
| --- | --- |

4. What is the number and size of the containers used if you use containers to garden?

| **Type of container** | **Number of containers** | **Approximate size of the container** | **Measurement unit**  **a) meters cubed**  **b) liter c) gallon** | **Additional information** |
| --- | --- | --- | --- | --- |
| Double-bottom from Alternatives |  |  |  |  |
| Double-botton made at home |  |  |  |  |
| Biotop |  |  |  |  |
| Rootpouch |  |  |  |  |
| Smartpot |  |  |  |  |
| Home-made bag |  |  |  |  |
| Plastic container (without water reservoir) |  |  |  |  |
| Other (specify) |  |  |  |  |

|  | **Mark the inputs that you use with an "x" .** | **How much input did you use?** | **Measurement unit a) kilogram; b) pounds; c) tonnes; d) meter cubed; e) liter; f) gallon; g) cubic yard** | **Where did the inputs come from 1) on-site; 2) Neighbor/friend; 3) Store; 4) Farmer or producer located on the island; 5) Municipality; 6) Other (specify)** | **What is the N :P :K* ratio (if you know it)?** | **Additional information** |
| --- | --- | --- | --- | --- | --- | --- |
| Potting mix |  |  |  |  |  |  |
| Fertilized potting mix (e.g. miracle grow mix) |  |  |  |  |  |  |
| Black soil |  |  |  |  |  |  |
| Clay-based soil |  |  |  |  |  |  |
| Peat |  |  |  |  |  |  |
| Perlite |  |  |  |  |  |  |
| Vermiculite |  |  |  |  |  |  |
| Coco fibers |  |  |  |  |  |  |
| Other soil/substrate used (specify) |  |  |  |  |  |  |
| Vermicompost |  |  |  |  |  |  |
| Shrimp and/or crab compost (or other marine based compost) |  |  |  |  |  |  |
| Bio-forest compost |  |  |  |  |  |  |
| Plant-based compost (green and table waste) |  |  |  |  |  |  |
| Other compost types used (specify) |  |  |  |  |  |  |
| Sheep/goat manure |  |  |  |  |  |  |
| Cow/beef manure |  |  |  |  |  |  |
| Chicken manure |  |  |  |  |  |  |
| Horse manure |  |  |  |  |  |  |
| Mixed source manure |  |  |  |  |  |  |
| Other manure types used (specify) |  |  |  |  |  |  |
| Solid fertilizer |  |  |  |  |  |  |
| Liquid fertilizer |  |  |  |  |  |  |
| Bone meal |  |  |  |  |  |  |
| Shrimp and/or crab meal (or other marine based meal) |  |  |  |  |  |  |
| Fish emulsion |  |  |  |  |  |  |
| Marine algae |  |  |  |  |  |  |
| Other fertilizer types used (specify) |  |  |  |  |  |  |
| Straw |  |  |  |  |  |  |
| Hay |  |  |  |  |  |  |
| Wood chips |  |  |  |  |  |  |
| Leaves |  |  |  |  |  |  |
| Non-organic materials |  |  |  |  |  |  |
| Other mulch types used (specify) |  |  |  |  |  |  |

Note: Biotop containers are about 30 liters, the Smartpot containers vary between 4 and 760 liters, the double-bottoms from Alternatives are about 70 liters, the Rootpouch bags vary between 3.8 and 2271liters.

QUESTIONS ABOUT INPUTS

5. The following table will allow you to answer the following three questions:

a) What are the inputs (soil and other substrates, compost, manure, fertilizer, and mulch) you used in the reference season you indicated at the beginning of the survey?

b) How much of each input did you use for the total area of all gardens in the same reference year?

c) Where did you get these inputs?

Note: Only fill out the sections that match your gardening practices.

*N:P:K is the ratio of nitrogen, phosphorus and potassium in your fertilizer or compost. This number is often written on the bag, or it is possible that you have done chemical analyses in lab on your compost or soil and thus know this ratio.

6. a) Do you use the following methods to get rid of your organic residues?

|  | **Yes** | **No** | **Additional information** |
| --- | --- | --- | --- |
| Composting your-self* |  |  |  |
| Left on soil |  |  |  |
| Municipal collection of green and food waste (to be composted) |  |  |  |
| Private company collection of green and food waste (to be composted) |  |  |  |
| Landfill |  |  |  |
| Other (specify) |  |  |  |

*Production of compost your-self includes: compost produced in a compost on-site or off-site with a partner on the island of Montreal. It can include garden waste, food waste, and high carbon materials like wood, leaves, cardboard and paper.

6. b) If you compost, how much compost do you produce?

|  | **Quantity in a year** | **Measurement unit a) kilogram;**  **b) pound;**  **c) cubic meter; d) liter; e) gallon;**  **f) cubic yard** | **Additional information** |
| --- | --- | --- | --- |
| Compost production |  |  |  |

Questions on food production and consumption

7. a) Do you measure food production (harvest) in your garden(s)?

| **Yes** | **No** | **Additional information** |
| --- | --- | --- |
|  |  |  |

7. b) How much did you harvest during the reference year?

|  | **Quantity harvested over the year** | **Measurement unit a) kilogram;**  **b) pound;**  **c) meter cubed; d) liter; e) gallon;** | **Additional information** |  |
| --- | --- | --- | --- | --- |
|  |  |  |  |  |
|  |  |  |  |  |
|  |  |  |  |  |
| Total |  |  |  |  |

8. a) Who consumes the fruits and vegetables produced in your garden(s) and in and in what quantity or proportion?

|  | **Mark all the groups that consume the fruits and vegetables harvested in your garden(s) with an "x****”.** | **Quantity** | **Measurement unit a) kilogram;**  **b) pound;**  **c) meter cubed; d) liter; e) gallon;**  **f) % of harvest** |
| --- | --- | --- | --- |
| Gardener(s) and their family and friends |  |  |  |
| Food bank |  |  |  |
| Employees (of your company) |  |  |  |
| Clients |  |  |  |
| Other (specify) |  |  |  |

8. b) If you sell all or part of **your (or your organization’s) harvest**, how to distribute it to your clients?

| **If you have clients….** | **Mark all the distribution circuits you use with an “x”** | **Quantity** | **Measurement unit a) kilogram**  **b) pound**  **c) meter cubed d) liter e) gallon**  **f) % of harvest** | **Additional information** |
| --- | --- | --- | --- | --- |
| Community supported agriculture baskets (CSA) |  |  |  |  |
| On-farm (garden) sale |  |  |  |  |
| Public market |  |  |  |  |
| Grocery store (through a whole saler) |  |  |  |  |
| Grocery store (directly) |  |  |  |  |
| Restaurants |  |  |  |  |
| Other (specify) |  |  |  |  |

Additional Information

If you have any additional information, comments, or suggestions about this survey please share them with us here. Also, if you know other urban agriculture actors (participants) that you believe would be relevant to this research (and this survey) please indicate them here.

|  |
| --- |

- 1. **Available inputs to gardeners, assumptions in calculating flows in the UA system, and possible yields in Montreal**

We visited the largest garden retailers on the island (in person and online) to document all inputs containing P (their dimensions, weight, and P content) in order to provide visual examples to survey participants, and also to calculate average densities and P concentrations for inputs when not provided by the participant. We did not include soil if survey participants did not have weight and P content information on-hand. Companies are not required by law to measure or report P content for soil blends and thus the majority do not measure P content.

Although we scaled-up P in fertilizer and compost from surveys to represent the total amount of P inputs to the area undercultivation in UA (see S1.3), we did not scale up flows related to animal production, and combined and omitted certain P flows in the system. Animal flows were used directly without scaling because we surveyed all large-scale farms with animals. We did not include losses through runoff and erosion, proportion of harvest that might be grown but eaten by animals in our system, or any primary data on biophysical measures. We combined recycled inputs (plant residues, compost, vermicompost, and animal manures) into one flow in order to maintain anonymity of survey participants.

When estimating yield, we used conservative yield estimates so as to avoid over estimating the amount of P harvested. When yield data was not available through our survey, we estimated yield based on data obtained in gardens in Montreal and New York City. We compared our estimated yield to other studies about UA and our own survey yield data to ensure our estimate was reasonable (Figure S1). Studies using data from many countries support the claim that smaller plots in rural areas can achieve higher yields than larger agricultural fields [2,3], as such yields for biodiverse production in small plots in urban areas can be assumed to be high (although yields may vary because of regional local biophysical conditions and management practices).

- 1. **Estimating total area in UA on the island of Montreal**

We used different methods for each type of UA: farms, collective gardens, community gardens, and private gardens as data sources were different, and then added them together in order to estimate the total area in UA production on the island.

**Farms**: Although we were able to survey the majority of farms on the island, two did not respond to our invitations. For the two farms missing from our surveys, and for which we therefore were lacking area information, area data was found on the *Agriculture Montréal* website [7], where farms, organizations, and individuals are invited to register the location and name of their garden into a map database and can include the area under cultivation (consulted January 14^th^ 2014)). By adding these two farms to those we surveyed we have considered the total area used by this type of UA in Montreal.

**Collective gardens**: Area data for collective gardens that we did not survey were found on the *Agriculture Montréal* website [7]. For those collective gardens that did not report an area under production but were listed on the website, we used the average area of all collective gardens that did report area (90.84m^2^).

**Community gardens**: The total area under production in community gardens was provided by the City of Montreal [8].

**Private gardens**: We used a City of Montreal survey as a basis of our estimate for the area under cultivation in private gardens, making different assumptions on garden size if gardens were on the round or on roofs and/or balconies. City data indicates that about 42 % of households participate in UA [9]. For those households practicing UA in yards (i.e., not on roofs or balconies) according to City data, we multiplied the number of households by the average area of a vegetable garden in the USA (56 m^2^ or 4% of the average American yard [10]). We chose to use the size of the average American yard garden as it was derived from a larger (and thus more robust) dataset, and this area is still comparable to what other smaller studies have found. For example, in Toronto, surveys indicated that the average vegetable and fruit garden in private yards was 41m^2^ [11], and that such private gardens could represent approximately 1% of the Toronto area [12]. In Chicago, mapping through Google earth and GIS revealed that home private gardens represented 3 times the area of community garden spaces, and that these private garden areas are often overlooked in estimates because they are hard to map [13]. Based on our estimates of private garden size, the area they occupy is approximately twice that of known community gardens, and about 2.7% of the total area of the island and is thus within the range of possibility. For those households practicing UA on their balcony or roof, we multiplied the number of households by the area of four *Alternatives* gardening containers (4*0.24m^2^, *Alternatives* containers are commonly used in Montreal and can be made at home from a city recycling bin). Four containers represents just under 1m^2^ which can fit on balconies in Montreal and represents the minimum space allocated by any of our survey participants that cultivated in containers, and is thus a conservative estimate of the area under cultivation on roofs and balconies.

1. LimeSurvey Project Team, Schmitz C (2012) LimeSurvey: An Open Source survey tool. Hamburg, Germany: LimeSurvey Project.

2. Cornia GA (1985) Farm size, land yields and the agricultural production function: an analysis for fifteen developing countries. World Development 13: 513-534.

3. Barrett CB, Bellemare MF, Hou JY (2010) Reconsidering conventional explanations of the inverse productivity-size relationship. World Development 38: 88-97.

4. Duchemin E, Wegmuller F, Legault A-M (2009) Urban agriculture: Multi-dimensional tools for social development in poor neighbourhoods. Field Actions Science Reports The journal of field actions 1.

5. Ackerman K (2011) The potential for urban agriculture in New York City: Growing capacity, food security, and green infrastructure. Columbia University, The Earth Institute, Urban Design Lab.

6. McClintock N, Cooper J, Khandeshi S (2013) Assessing the potential contribution of vacant land to urban vegetable production and consumption in Oakland, California. Landscape and Urban Planning 111: 46-58.

7. Collectif de recherche en aménagement paysager et agriculture urbaine durable (CRAPAUD), Institut des sciences de l'environnement de l'Université du Québec à Montréal, Laboratoire sur l’agriculture urbaine (AU/LAB) (2013) Vitrine de l’agriculture urbaine à Montréal. Montréal.

8. Office de consultation publique de Montréal (2012) État de l’Agriculture Urbaine à Montréal - Rapport de consultation publique. Montréal, Québec, Canada: Office de consultation publique de Montréal. pp. 157.

9. Ville de Montréal (2013) Sondage auprès de la population de l’Île de Montréal sur l’agriculture urbaine (Sommaire exécutif). In: Montréal DdddVd, editor. Montréal.

10. Butterfield B (2009) The impact of home and community gardening in America. South Burlington, VT. Accessed February.

11. Kortright R, Wakefield S (2011) Edible backyards: a qualitative study of household food growing and its contributions to food security. Agriculture and Human Values 28: 39-53.

12. MacRae R, Gallant E, Patel S, Michalak M, Bunch M, et al. (2010) Could Toronto provide 10% of its fresh vegetable requirements from within its own boundaries? Matching consumption requirements with growing spaces. Journal of Agriculture, Food Systems, and Community Development 1: 105-127.

13. Taylor JR, Lovell ST (2012) Mapping public and private spaces of urban agriculture in Chicago through the analysis of high-resolution aerial images in Google Earth. Landscape and Urban Planning 108: 57-70.
